# Selective forces and mutational biases drive stop codon usage in the human genome: a comparison with sense codon usage

**DOI:** 10.1186/s12864-016-2692-4

**Published:** 2016-05-17

**Authors:** Edoardo Trotta

**Affiliations:** Institute of Translational Pharmacology, Consiglio Nazionale delle Ricerche (CNR), Rome, 00133 Italy

**Keywords:** Stop codon, Codon bias, Human genome, GC content, Transcriptome, Minimum folding energy

## Abstract

**Background:**

The three stop codons UAA, UAG, and UGA signal the termination of mRNA translation. As a result of a mechanism that is not adequately understood, they are normally used with unequal frequencies.

**Results:**

In this work, we showed that selective forces and mutational biases drive stop codon usage in the human genome. We found that, in respect to sense codons, stop codon usage was affected by stronger selective forces but was less influenced by neutral mutational biases. UGA is the most frequent termination codon in human genome. However, UAA was the preferred stop codon in genes with high breadth of expression, high level of expression, AT-rich coding sequences, housekeeping functions, and in gene ontology categories with the largest deviation from expected stop codon usage. Selective forces associated with the breadth and the level of expression favoured AT-rich sequences in the mRNA region including the stop site and its proximal 3’-UTR, but acted with scarce effects on sense codons, generating two regions, upstream and downstream of the stop codon, with strongly different base composition. By favouring low levels of GC-content, selection promoted labile local secondary structures at the stop site and its proximal 3’-UTR. The compositional and structural context favoured by selection was surprisingly emphasized in the class of ribosomal proteins and was consistent with sequence elements that increase the efficiency of translational termination. Stop codons were also heterogeneously distributed among chromosomes by a mechanism that was strongly correlated with the GC-content of coding sequences.

**Conclusions:**

In human genome, the nucleotide composition and the thermodynamic stability of stop codon site and its proximal 3’-UTR are correlated with the GC-content of coding sequences and with the breadth and the level of gene expression. In highly expressed genes stop codon usage is compositionally and structurally consistent with highly efficient translation termination signals.

**Electronic supplementary material:**

The online version of this article (doi:10.1186/s12864-016-2692-4) contains supplementary material, which is available to authorized users.

## Background

The nucleic acid unit of the genetic code is a word of three nucleotides, termed codon. The genetic code consists of 64 codons: 61 sense codons, that code for 20 amino acids; and 3 stop codons, UAA, UAG and UGA, that signal the termination of coding sequence. In 20 human selenoprotein genes, the stop codon UGA also encodes the amino acid selenocysteine [1] by a reassignment mechanism that is directed by a specific stem-loop structure located in the 3’ untranslated region (3’ UTR) [1, 2]. The stop signal and almost all the amino acids are coded by more than one codon, termed synonymous codons, which are used with unequal frequencies. The causes of the uneven use of synonymous codons, named codon usage bias or codon bias, are not yet fully understood. In human genome, the GC-content of contiguous coding and non-coding regions correlates significantly [3] in agreement with a role of neutral mutational biases in codon usage [4]. The influence of selection on codon usage in the human genome is not well-defined [5–8]. Codon usage is not significantly biased in highly expressed genes encoding ribosomal proteins and histones [9, 10] and is not significantly correlated with protein abundance [8], even if studies report a weak correlation of mRNA abundance with the codon bias index and with codons associated with the highest tRNA gene copy number [8, 11].

Most of the studies investigating the causes of codon bias in the human genome have been focused on the analysis of sense codons, generally neglecting any reference to stop codon usage [5–8, 11]. Stop and sense codons are characterized by different recognition mechanisms. Whereas sense codons are recognized by specific aminoacyl-tRNAs, stop signals are the target of proteins called release factors. In eukaryotes, the three stop signals are recognized by the same tRNA-shaped protein, the release factor eRF1 [12, 13]. Moreover, the stop codon site marks the boundary of the protein coding region with the 3’ UTR, which contains regulatory motifs affecting the translation, localization, and stability of transcripts. These differences between stop and sense codons could be reasonably reflected in different selective forces influencing their usage.

In higher eukaryotes the most frequent stop codon is UGA, UAA is used mostly in lower organisms, and UAG is used least frequently in all eukaryotes [14]. No correlation of stop codon bias with GC3 or gene expression level has been found within individual eukaryotic genomes [14], even if stop codon usage is correlated with transcription levels in yeast [15], and the UGA and UAA stop codons appear to be the most used codons in low and highly expressed genes of mammals, respectively [16]. The identity of the stop codon and of the flanking base at the downstream position (+1) have been associated with the efficiency of translational termination. UAA is the most efficient terminator [17] and the order for termination efficiency of the base at +1 position was found to be A ≈ G> > C ≈ U, independently of the stop codon, UAAA being the most efficient four-base combination [18]. Moreover, relaxed secondary structures, which may facilitate the termination of translation, have been associated with stop codon sites [19] and the UAA stop signal appears to be generally characterized by local loop structure while UGA does not exhibit significant structural preferences [14].

This work is an analysis of stop codon usage in the human genome using sense codons as the element of comparison. Here, we investigate the relationship of stop codon usage with a series of genomic properties: the GC-content of coding sequences (CDSs), the level and breadth of gene expression, chromosomal location, gene function, and the sequence and structural contexts of the stop sites. The results show that both neutral mutation and selective forces influence stop codon usage in the human genome.

## Results

### The relative frequencies of synonymous stop and sense codons coherently correlate with the GC-content of coding sequences

**Fig. 1:**
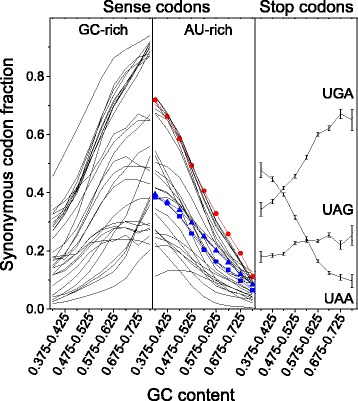
Synonymous codon fraction versus GC content. Relative frequencies of stop (right panel), GC-rich (left panel), and AU-rich (middle panel) synonymous codons in gene categories with increasing GC content. Red circles indicate the fraction of AU-rich codon (UGU) of the 2-fold degenerated cysteine family (UGC and UGU). Blue symbols indicate the fraction of AU-rich codons (GCA and GCU) of the 4-fold degenerate alanine family (GCA, GCC, GCG and GCU). Values in the right panel represent mean ± SEM

**Fig. 2:**
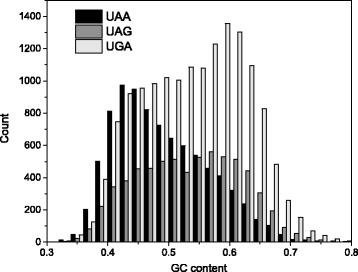
Frequency distribution of GC content. The three diagrams show GC content distribution in the sets of coding sequences containing UAA (black bars), UAG (grey bars), and UGA (white bars) stop codons

To evaluate the influence of mutational bias on stop codon usage in the human genome, we performed a comparative analysis between the use of stop and sense codons in relation with the GC-content of CDSs. The study was performed by clustering 30497 human consensus CDSs in categories of GC-content (step = 10 %) and by computing the relative frequencies of the synonymous codons for each GC category. The results are summarized in Fig. 1. As shown in the figure, the fraction of each synonymous codon changes consistently and monotonically with the GC content of coding sequences. As a general rule, with increasing the GC-content of CDSs, the codons that are richer in GC within their synonymous family increase their fraction and, accordingly, the occurrence of those richer in AU decreases. This is true for both sense and stop codons, showing that the stop site is affected by the same mutational processes as sense codons (Fig. 1). It should be noted that, with increasing GC-content, the relative frequency of UAG grows significantly less than UGA. The correlation between the nucleotide composition of CDSs and the stop codon usage is also evident from the frequency distribution of the GC-content in the three sets of CDSs containing UAA, UAG and UGA stop codons (Fig. 2). It should be noted that the frequency distributions of GC content in the three sets of CDSs reported in Fig. 2 show a bimodal (UAG) or a skewed (UAA and UGA) shape. These distribution shapes are consistent with the relationship of stop codon usage with GC3 that in human genome exhibits a bimodal distribution [20].

From CDSs with lowest to highest GC-content, the fraction of sense codons that within their synonymous family are richer in AU decreases on average by 0.60. For example, in cysteine family UGU fraction decreases from 0.72 to 0.11, and in alanine family the AU-rich codons GCA and GCU decrease overall by 0.63 (Fig. 1). In the same GC range, the fraction of AU-rich stop codon UAA decreases by just 0.38. Since neutral forces associated with genomic GC-content should act equally on sense and stop codons, the scarce variation of stop codon fraction with GC-content suggests the action of additional forces that partially contrast mutational effects on stop codons.

### UAA is statistically frequent in 20 gene ontology categories incorporating ribosomal genes

As reported above, both sense and stop codon usage in the human genome correlate with the GC-content of CDSs. However, the correlation analysis appears consistent with a significantly weaker effect of mutational biases on stop sites than on sense codons. This difference highlights the distinct roles of the two classes of codons and, notably, suggests that the codon preference at the stop site may be influenced by additional forces that are weaker at the sense codon sites. It has been reported that sense codon usage in the human genome is correlated with gene function [21]. Therefore, to test the possibility that stop codon usage could be related with the function of the expressed proteins, we computed the relative frequencies of stop codons in gene ontology (GO) categories [22]. To identify anomalous distributions of stop codons within GO categories, we tested the null hypothesis that the frequency of each stop codon in a GO category follows a Poisson binomial distribution. The occurrence of a stop codon in a gene is assumed to be a Bernoulli trial with the probability estimated from its fraction in the genomic CDSs with the same GC content (see Methods section). The results should emphasize those GO categories that display stop codon frequencies not predictable by the GC-content of their CDSs.

**Fig. 3:**
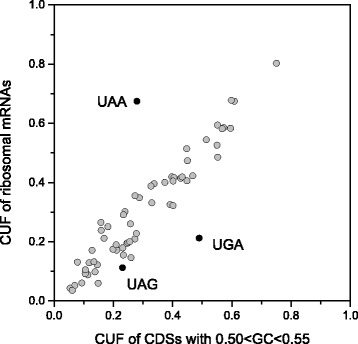
Comparison between codon usage frequency (CUF) of ribosomal genes and coding sequences (CDSs) with 0.50 < GC < 0.55. Stop and sense codons are indicated by black and grey circles, respectively

The strongest deviations from the expected stop codon frequencies were found for 20 GO categories that preferentially use UAA. These 20 GO terms, listed in Additional file 1: Table S1, are generally associated with processes that involve protein-mRNA complexes and, surprisingly, all of them include genes coding ribosomal proteins. In fact, the category of ribosomal mRNAs present an extremely high preference for UAA (67.5 %, *N* = 80). It is interesting to observe that if the ribosomal proteins were discarded from the whole dataset, all the UAA-rich GO categories maintained a level of UAA (ranging from 32 to 47 %) higher than that of the global genome (28 %). Remarkably, with respect to the genes of the same GC category, the ribosomal mRNAs exhibit a significant high frequency of UAA, but no differences in the usage of sense codons (Fig. 3). In fact, the average GC-content in the CDSs of ribosomal mRNAs (51.4 %) is quite similar to the average over all CDSs (52.0 %) whereas the UAA/non-UAA ratio in the stop site is strongly different: 2.08 for ribosomal mRNAs and 0.39 for all CDSs. Therefore, the high frequency of UAA in ribosomal mRNAs does not appear associated with the neutral mutational processes that generally affect both the stop and sense codon usage, but with forces that act selectively on stop codons, with scarcely detectable effects on sense codon usage.

In summary, only a limited number of GO categories present a stop codon bias statistically different from those expected from the GC-content of their CDSs. The most significant stop codon biases were the high frequencies of UAA in 20 GO categories that share ribosomal proteins and are related to protein-RNA complexes.

### Transcription breadth is more correlated with stop than with sense codon usage

**Fig. 4:**
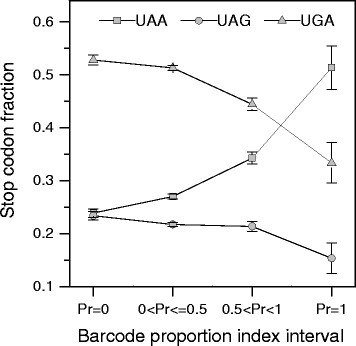
Change of stop codon fractions with the breadth of transcription. Transcriptome data were obtained from the Gene Expression Barcode (Barcode) database. Genes were clustered into four categories by proportion of cell types in which a gene was estimated as expressed (Pr). Values represent mean ± SEM

Selective forces weakly influence codon bias in the human genome [5–7]. It has been reported that, in the human genome, sense codon usage is more correlated with the breadth than with the level of expression [5] and that the broadly expressed genes cluster in regions of high GC content [23]. To test the hypothesis that the stop codon preference could be related with the breadth of expression, we used transcriptome data from the Gene Expression Barcode (Barcode) database [24] to estimate the proportion (Pr) of cell or tissue types in which a given gene can be identified as expressed (see Methods). In our analysis, genes were clustered into four categories with increasing breadth of expression on the basis of their Pr value: Pr = 0, 0 < Pr ≤ 0.5, 0.5 < Pr < 1, and Pr = 1. Figure 4 reports the relative frequency of stop codons for each of the four expression categories. As shown in the figure, transcription breadth correlates positively with UAA fractions and negatively with UGA and UAG frequencies. Pairwise comparison shows that the differences in stop codon fractions among the first (Pr = 0) and the last (Pr = 1) gene expression categories are statistically significant for all the three stop codons (*t*-test *p*-value < 0.05). Moreover, using a Poisson binomial model (see Methods section), we found that only the gene categories with high breadth of expression exhibited a significant deviation of stop codon frequencies from those expected by the GC-content of their CDSs (Table 1).

**Table 1:** P-values for the stop codon frequencies in four Gene Expression Barcode categories

Gene Expression Barcode category	p(x ≤ n.TAA)	p(x ≥ n.TAA)	p(x ≤ n.TAG)	p(x ≥ n.TAG)	p(x ≤ n.TGA)	p(x ≥ n.TGA)
Pr = 0	0.2063	0.8067	0.8275	0.1844	0.4802	0.5354
0 < Pr ≤ 0.5	0.2502	0.7571	0.1865	0.8199	0.9150	0.0881
0.5 < Pr < 1	0.9994	7.03E-04	0.5168	0.5061	1.55E-03	0.9987
Pr = 1	1.0000	1.07E-08	5.00E-02	0.9684	4.61E-05	1.0000

Genes were clustered into four categories by proportion of cell types in which a gene was estimated as expressed (Pr). P-values were computed on the basis of Poisson binomial cumulative distribution. The probability p of codon occurrence in each gene was set equal to the codon fraction expected at the GC-content of its CDS

**Fig. 5:**
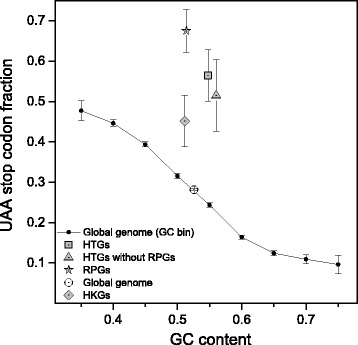
UAA stop codon fraction versus GC-content of coding sequence. Black circles connected by lines indicate the UAA fractions in GC content categories of genomic CDSs. Single symbols indicate the average UAA fraction and GC content of highly transcribed genes (HTGs, grey square), ribosomal protein genes (RPGs, grey star), housekeeping genes (HKGs, grey diamond), HTGs without RPGs (grey triangle), and global genome (open circle). Values represent mean ± SEM

Housekeeping genes encode proteins involved in basic cellular processes that are characterized by a high breadth of expression. Thus, we verified the preference of UAA in genes with a high breadth of expression by analysing a further independent source of genes annotated as housekeeping. Because the identity of housekeeping genes is not uniformly identified by the various sources reported in the literature [25], we used a set of 62 genes selected as highly shared among 15 different housekeeping gene lists [25]. As illustrated in Fig. 5, the results show a high preference of housekeeping genes for UAA (UAA fraction = 0.452) that is not statistically consistent with the average GC level of their coding sequences (one-sample *t*-test *p*-value < 0.05).

**Fig. 6:**
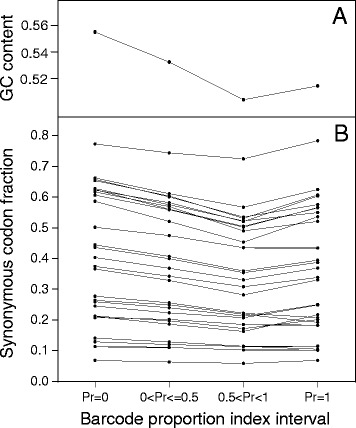
GC content **a** and fraction of GC-rich synonymous codons **b** in gene categories with increasing breadth of transcription. Transcriptome data were obtained from the Gene Expression Barcode (Barcode) database. Genes were clustered into four categories by proportion of cell types in which a gene was estimated as expressed (Pr)

We have shown above, that the changes in codon usage associated with the variation of GC-content are weaker in stop than in sense codons (Fig. 1). If we compare the changes of stop and sense codon usage with transcription breadth the result is inverted; clear change in the relative frequencies of stop codons (Fig. 4) is concomitant with an almost constant fraction of synonymous sense codons (Fig. 6b). We also measured the average GC-content of CDSs in the four gene expression categories. We found that GC-content decreases with breadth of transcription within the range 0 ≤ Pr < 1 and then increases in the last category with Pr = 1 (Fig. 6a). It is interesting to observe that the relative frequency of all GC-rich sense codons display the same curve-trend as GC-content (Fig. 6). This strongly suggests that the very weak relationship between the sense codon usage and the level of transcription is essentially a consequence of the relationship between GC-content and gene expression. In contrast with sense codons, stop codons change monotonically their synonymous fraction with transcription breadth (Fig. 4) indicating that, in the category of genes with the highest expression breadth (Pr = 1), the stop codon choice is mainly driven by forces not associated with mutational biases. These not neutral forces, acting selectively on termination site, may interfere with the correlation between GC-content and stop codon usage, explaining why stop codon usage changes less than sense codon usage with GC-content (Fig. 1).

### Highly expressed genes show a significant preference for the UAA stop codon

It is argued that, in eukaryotic genomes, gene expression level does not correlate significantly with stop codon usage [14], even though, in yeast, stop codon usage is found to correlate with transcription level and specific stop codons are associated with mammalian genes that are expressed at different levels [15, 16]. Consistently with the high frequency of UAA in highly expressed genes [16], as stated above, we have shown an anomalously high preference of UAA in the class of highly expressed ribosomal genes. To check the presence of a relationship between stop codon preference and gene expression at a genome-wide level, we analysed the relationship between stop codon usage and transcription level using a source of human transcriptome data taken from recent literature [26]. From this dataset, concerning the 100 most expressed genes for each of 26 tissues, we selected a subset of 62 genes that were shared by at least seven tissues. As illustrated in Fig. 5, the UAA fraction of this set of highly expressed genes (UAA fraction = 0.565) is significantly higher than expected from their GC-content (UAA fraction = 0.242, GC = 0.5475). The UAA fraction remains high (UAA fraction = 0.515) even after discarding the ribosomal genes from the dataset (Fig. 5).

### The distribution of the three stop codons among chromosomes is correlated with the average chromosomal GC-content of coding sequences

**Fig. 7:**
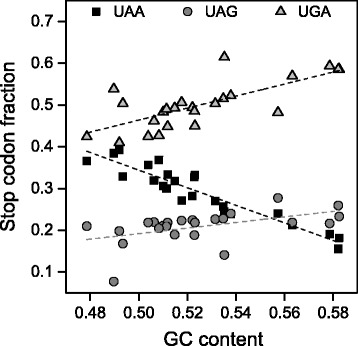
Scatter plot of stop codon fractions versus GC content of coding sequences (CDSs) in chromosomes. Broken lines indicate linear regressions

For each chromosome we computed the average GC content of its CDSs and the synonymous stop codon fractions. As shown in Fig. 7, the computed GC-content of chromosomes is strongly correlated with the relative frequencies of UAA (Pearson’s correlation coefficient (Rp) = −0.94, *p* < 0.05), UAG (Rp = 0.50, *p* < 0.05), and UGA (Rp = 0.73, *p* < 0.05). It is interesting to observe that the GC content accounts for about 88 % of the variation of UAA fraction in chromosomes (coefficient of determination (R^2^) = 0.88).

We extended the chromosome analysis to the study of the relationship between stop codon usage and the breadth of transcription using the Pr index of Barcode database. The results indicate that the average breadth of gene transcription in chromosomes is significantly correlated with the chromosome fraction of UAA (Rp = 0.619, *N* = 23, *p* < 0.05), UGA (Rp = −0.53, *N* = 23, *p* < 0.05). Therefore, the distribution of stop codons among chromosomes is correlated with the GC-content of coding sequences and with the breadth of transcription. The relationship of the breadth of transcription with stop codon usage could be partially due to its correlation with GC content. In fact, we found that in chromosomes the breadth of transcription and GC content are strongly correlated (Rp = −0.67, *N* = 23, *p* < 0.05). When the variation of GC-content was partialled out, the relationship of Pr with UAA and UGA fraction disappears (partial correlation: Rp = −0.02 and 0.035, respectively) suggesting a non-causal relationship between the breadth of transcription and stop codon usage.

### The minimum folding energy of mRNA regions surrounding the stop site is correlated with stop codon usage

**Fig. 8:**
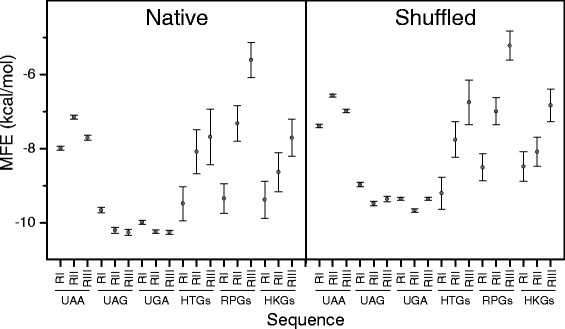
The minimum folding energy of mRNA regions surrounding the stop site. Minimum folding energy (MFE) of region I (RI), II (RII), and III (RIII) in mRNAs containing UAA, UAG, UGA, and in highly transcribed (HTG), housekeeping (HKG), and ribosomal protein (RPG) genes. Sequences are 50 nucleotides long. RI contains the coding sequence flanking the 5’ end of the stop codon (stop codon not included), RII is centred on the stop codon, and RIII is the 3’-UTR sequence flanking the stop codon (stop codon not included). Panels A and B report the average MFE of native and shuffled sequences, respectively. Values represent mean ± SEM

In eukaryotes, loop structure is more associated with UAA than with UGA or UAG stop codons [14] and, in general, stop codon regions are characterized by relaxed secondary structures which may facilitate termination of translation [19]. It is also reported that mRNA secondary structures affect translation efficiency [27–29]. During active translation, each mRNA is simultaneously translated by a cluster of ribosomes that are linearly distributed along the coding sequence, forming a multimolecular complex termed polysome. Because of the unwinding action of ribosomes, mRNA is allowed to fold into stem-loop structures only locally [27]. To investigate the relationship between stop codon usage and local secondary structures, we computed the minimum folding energy (MFE) of three 50 nt-long overlapping regions localized in the area of the stop signal that for clarity we define as region I, II and III. Region I contains the coding sequence flanking the 5’ end of the stop codon (stop codon not included), region II is centred on the stop codon, and region III is the 3’-UTR sequence flanking the stop codon (stop codon not included). Figure 8 summarizes the predicted MFE of the three regions in the sets of mRNAs containing UAA, UAG and UGA stop codons. As shown in the figure, the structures of the three regions associated with UAA are significantly less stable than those related to UAG and UGA.

### The low structure stability associated with UAA is merely attributable to the nucleotide composition, rather than to the nucleotide order in the sequence

In the previous paragraph, we showed that three 50 nt-long overlapping regions localized in the area of the stop signal associated with UAA are significantly less stable than those related to UAG and UGA. Such a different folding stability may originate from differences in their nucleotide sequence or composition, or from a combination of the two causes. To eliminate the contribution of the nucleotide sequence and establish the role of the nucleotide composition, the nucleotide order of each analysed sequence was shuffled 100 times. As shown in Fig. 8, all shuffled sequences exhibit an average free energy slightly higher than that of their native sequences, consistently with the reported evidence that native mRNAs have a more stable structures than their shuffled forms [30]. However, the shuffling procedure does not affect the differences among the three stop codons showing that the relatively low structure stability associated with UAA is merely attributable to the nucleotide composition rather than to the nucleotide order in the sequences.

### Base composition strongly destabilizes local secondary structures in region III of ribosomal messengers

**Fig. 9:**
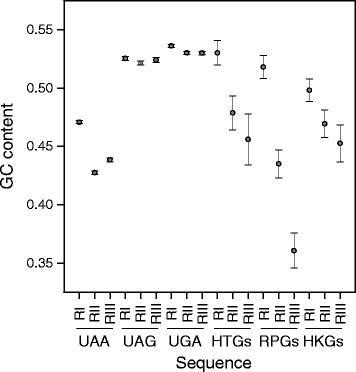
The GC content of mRNA regions surrounding the stop site. GC content of region I (RI), II (RII), and III (RIII) in mRNAs containing UAA, UAG, UGA, and in highly transcribed (HTG), housekeeping (HKG), and ribosomal protein (RPG) genes. Sequences are 50 nucleotides long. RI contains the coding sequence flanking the 5’ end of the stop codon (stop codon not included), RII is centred on the stop codon, and region RIII is the 3’-UTR sequence flanking the stop codon (stop codon not included). Values represent mean ± SEM

In ribosomal messengers, the computed MFE of the three regions are anomalously different from each other (Fig. 8). In particular, ribosomal mRNA exhibits a considerable and progressive decrease of local folding stability from region I to region III, consistent with a significant reduction of GC-content (Fig. 9) and resulting in a remarkably GC-poor and structurally unstable region III. A progressive decrease from 5’ to 3’ direction of both the average folding stability and the GC-content was also observed in the termination sites of genes with high breadth and high level of expression (Figs. 8 and 9). It appears that, in region II and especially in region III, selection strongly favours base compositions that destabilize local secondary structures.

### Base usage at +1 position is correlated with both the GC content of coding sequences and the breadth of gene expression

**Fig. 10:**
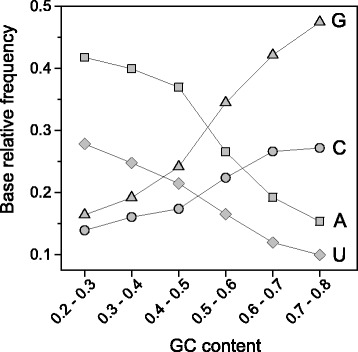
Change of base relative frequencies at +1 position in gene categories with increasing GC content

**Fig. 11:**
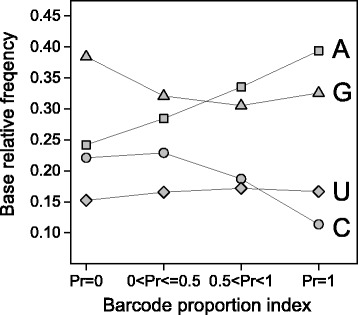
Change of base relative frequencies at +1 position in gene categories with increasing breadth of transcription. Transcriptome data were obtained from the Gene Expression Barcode (Barcode) database. Genes were clustered into four categories by proportion of cell types in which a gene was estimated as expressed (Pr)

**Fig. 12:**
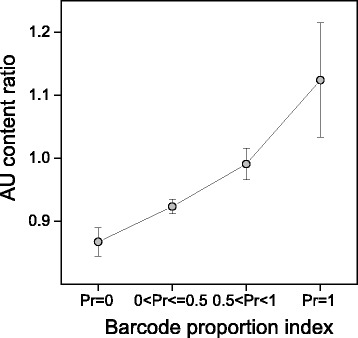
Ratio between A + U occurrence at +1 site and region I in gene categories with increasing breadth of transcription. Transcriptome data were obtained from the Gene Expression Barcode (Barcode) database. Genes were clustered into four categories by proportion of cell types in which a gene was estimated as expressed (Pr). Values represent mean ± SEM

In mammals, as well as in other organisms, the nucleotide context flanking the stop codons is an important factor influencing the efficiency of translational termination. In particular, the downstream base flanking the stop codon (+1 position) can change the termination efficiency by 70-fold following UAA and 8-fold in the case of UGA and UAG [18]. We analysed the relationship of base usage at +1 position with the GC-content of region I and with the breadth of transcription estimated by the Pr index of Barcode database. The results, presented in Figs. 10 and 11, show that base usage at +1 position correlates with both the GC-content and the breadth of transcription. In particular, the occurrence of both C and G at +1 site correlates positively with the GC-content of region I, whereas the fractions of A and U correlate negatively. The change of the nucleotide usage at +1 position with GC-content appears considerable, especially for purines (0.16 ≤ G ≤ 0.47 and 0.42 ≤ A ≤ 0.15) (Fig. 10). As shown in Fig. 11, increasing the breadth of transcription is associated with a significant increase of A + U occurrence at +1 position. It should be noted that A and G are largely the most frequent bases in genes with the maximum breadth of expression (Pr = 1). Accordingly, we found a high occurrence of A (87 %) at +1 site in the class of ribosomal mRNAs. In addition, for each increasing barcode category we computed the ratio between the average A + U content at +1 sites and the average A + U content in regions I. The results, illustrated in Fig. 12, show that, with increasing breadth of transcription, A + U occurrence at +1 sites increases more steeply than in the last region of coding sequences, suggesting that selection associated with the breadth of expression influences the +1 site more than sense codon region.

## Discussion

There are differences among the usage of the three stop codons in eukaryotes: UAA is the most frequent in lower organisms, UGA is prevalent in higher organisms, and UAG is the least used in all eukaryotes [14]. Genomic complexity and GC-content are thought to contribute to the observed differences among different eukaryotes [14]. However, within genomes of single eukaryotic organisms, the results are confusing. It has been reported that stop codon usage shows no relationship with GC3 content and with gene expression level [14]. In contrast, it is shown that UAG and UAA exhibit a preference for low and highly expressed genes, respectively [16], and that stop codon usage is correlated with mRNA abundance in the yeast genome [15].

In contrast with the lack of any relationship between stop codon usage and GC3 previously reported [14], the results of the present work show that the relative frequencies of stop codons are significantly correlated with the GC-content of coding sequences (Figs. 1 and 7). In a set of 30492 human consensus CDSs, the relative frequency of UAA monotonically decreases from 0.48 to 0.10 in CDS categories with increasing GC-content. Conversely, the GC-rich stop codons UGA and UAG progressively increase their relative occurrences from 0.34 to 0.66 and from 0.18 to 0.25, respectively. We found that changes in the relative frequency of stop codons with GC-content is more modest than that averagely displayed by sense codons (Fig. 1), suggesting the presence of forces that, in contrast with mutational effects, act on stop codons more strongly than on sense codons. These contrasting forces could be related with selection associated with gene expression level. In fact, we found that, with increasing breadth of transcription, the fractions of synonymous stop codons change significantly more than those of sense codons. In categories with increasing breadth of transcription, the UAA fraction strongly increases from 0.24 to 0.51 (Fig. 4) whereas the change of sense codon usage appears weakly detectable (Fig. 6). Consistently, the ribosomal genes and an independent set of housekeeping genes exhibit normal sense codon usage but an anomalous high preference for UAA stop codon (Figs. 3 and 5).

It has been reported that translation termination is primarily directed by four bases, including the stop codon and its 3’-flanking base [31], with an efficiency that can increase up to 70 fold by a change in the single base 3’-adjacent to the stop codon [18]. Moreover, independently of the stop codon, the termination efficiency for A and G base at +1 position was found to be significantly higher than for C and T, UAAA being the most efficient four base combination [18]. The results of our work show that nucleotide usage at +1 position is correlated with both the breadth of transcription and GC-content (Figs. 11 and 10, respectively). Although the most prominent changes are associated with the GC-content, with increasing breadth of expression, we found a clear increase of an A preference at +1 position that is independent from the CDS GC-content. This increase of A frequency is concomitant with an increase of G frequency in the category with the highest transcription level. As a result, A and G are largely the most frequent bases at +1 site of the genes with the highest breadth of expression (Pr = 1) (Fig. 11). Since these two bases present the highest efficiency of termination [18], our result suggest a causal relationship between the nature of the four base termination site and the breadth of transcripts in the cell, accordingly with the need of the most ubiquitous transcripts to rely on a more efficient translation termination signal.

It is also reported that, in eukaryotes, UAA is more associated with loop structures than UGA and UAG [14]. In agreement, our results show that UAA is associated with local contexts, including the 50 nt long downstream and upstream sequences, significantly more weakly structured than those associated with UAG and UGA (Fig. 8). In addition, we show that the relatively labile local structures associated with UAA are merely attributable to the nucleotide composition rather than to the nucleotide sequence of the termination sites, as deduced from the small MFE differences between native and shuffled sequences (Fig. 8). Therefore, in general, the preference for UAA appears to reflect the compositional bias of a more extended region that includes the coding sequence and 3’-UTR. Thus, neutral mutational bias appears to be the most effective cause of the relationship between UAA and the labile structure of the termination sites. However, we found that UAA is favoured not only in GC-poor CDSs by neutral mutational bias, but also in highly expressed genes by selective forces. In this last case, selection acts by favouring A + U bases at stop sites and region III, but has almost no effect on sense codons. This compositional difference is reflected in significantly different local structural stabilities between the upstream and downstream regions of the stop codons. The compositional and structural difference between stop site and coding sequence excludes that the labile structures associated with the selection of UAA in highly expressed genes are favoured by processes that act directly on sense codons. For instance, it is not reasonable that the labile structures associated with UAA are favoured to increase translation speed by reducing ribosome pausing [32] because, in this case, we should observe the same structural effects on coding region. Conversely, it is reasonable that the preference of relaxed structures at stop sites of highly expressed mRNA could be related to their properties to promote more efficient termination signals [19]. Moreover, we cannot exclude that the sequence and structural properties of stop codon and region III may be related to post-transcriptional control of mRNA trafficking and metabolism. By favouring more relaxed structures, the AU preference may facilitate the accessibility and the recognition of trans-acting factors such as RNA-binding proteins and non-coding RNAs. In fact, the high use of A and U bases is a common characteristic of cis-regulatory elements of the 3’-UTR such as AREs (AU-rich elements) [33], CPEs (cytoplasmic polyadenylation elements) [34] and PASs (polyadenylation signals) [35]. Our results also suggest that the level and the breadth of expression could play an additive role on stop codon bias. In fact, the characteristic trait, induced by selection on the termination site of highly expressed genes, is emphasized in the ribosomal mRNAs that are characterized by high level and breadth of expression.

Lastly, we have analysed the stop codon distribution in genes clustered on the basis of chromosome location and GO terms. Synonymous stop codons are differently distributed among chromosomes, for example, the UAA fraction ranges from 0.155 in chromosome 22 to 0.392 in chromosome 18 (Fig. 7). About 88 % of the variation of UAA fraction in chromosomes is explained by the GC-content of CDSs, whereas no direct relationship, independent from GC-content, was detected between stop codon usage and gene expression. Therefore, the unequal distribution of stop codons in chromosomes is prevalently attributable to neutral mutational biases rather than selective forces associated with the breadth of expression.

With some exceptions, the frequency of stop codons in the functional categories defined by GO terms is mainly that expected by a random distribution. The most relevant exception is the statistically high preference for UAA in 20 GO categories that are related to protein-RNA complexes and share ribosomal proteins (Additional file 1: Table S1). It should be observed that the typical compositional and structural context associated with stop codon sites of the highly expressed genes described here are strongly emphasized in ribosomal genes making the mRNA regions covering the stop codons of this class of proteins extremely unusual. This peculiarity is remarkable because ribosomal biogenesis is directly involved in pathways of a number of human diseases and cancers [36]. For example, perturbation in ribosomal biogenesis leads to the stabilization and activation of p53 through the interaction of ribosomal proteins with MDM-2 [37].

## Conclusions

In summary, this study shows that both neutral mutation and selective forces influence codon usage in the human genome. The effects of mutational biases are more consistent on sense than on stop codons, whereas selective forces affect stop codons much more than sense codons. UAA is the preferred stop codon of genes with GC-poor CDSs and with the most abundant and most ubiquitous mRNAs. Selection associated with the breadth and the level of expression favours UAA and AU-rich proximal 3’-UTRs. Therefore, the stop codon usage is always coherent with the base composition of its 3’-proximal UTR, but not with its proximal 5’-coding region that is very weakly influenced by selective forces. The strong relationship between stop codon identity and base context of the proximal 3’-UTR can be explained by a common structural function related with the efficiency of translation termination, or with functional activities connected with processes involving mRNA metabolism.

## Methods

### Genome-wide datasets

Coding sequences were downloaded from the consensus CDS database (CCDS; release 17; ftp://ftp.ncbi.nlm.nih.gov/pub/CCDS/) [38], which provides high-quality human CDS data. Coding sequences with in-frame internal UGA (coding for selenocysteine) were discarded and only one copy of the CDSs shared between chromosomes X and Y were considered. The final set of CCDSs comprised 30492 sequences.

Upstream and downstream regions of stop codons were extracted from mRNA sequences of the Reference Sequence (RefSeq) collection downloaded from the National Center for Biotechnology Information (NCBI; http://www.ncbi.nlm.nih.gov) [39].

We downloaded the data reporting the association of proteins with GO terms and the interconnecting relationships among GO terms from the web server of Gene Ontology Consortium (http://geneontology.org). In the protein-GO term association we included ancestors with relationship annotated as is_a and part_of.

Cross reference data protein-CCDS were downloaded from Uniprot web server (http://www.uniprot.org/). Protein entries with more than one CCDS id were represented by only one stop codon if the CCDS entries contained the same stop codon otherwise were discarded. Overall, 93.4 % of the proteins (17074) were associated with one stop codon.

Transcriptome data for expression breadth analysis were downloaded from the Gene Expression Barcode web server (http://barcode.luhs.org; gene catalog: HGU133plus2 v3) [24]. Gene catalogs of Barcode webserver include the values of the proportion (Pr) of cell or tissue types in which a specific probe set is identified as expressed. In this work, to asses the proportion of cell or tissue type in which a specific gene is identified as expressed, probe sets annotated with high average entropy were discarded and, in cases of genes mapped by more than one probe, the highest proportion value were considered [40]. The results obtained from cell and tissue types were very similar, and the differences were irrelevant to the objective of this study. For this reason, we reported only results from cell data.

The list of highly transcribed genes (HTGs) was taken from recent literature [26], using genes annotated as the most expressed genes in tissues. From this dataset we selected a set of 62 genes that were shared by at least seven tissues.

GC-content and codon usage of ribosomal CDSs was determined using a dataset of 80 sequences downloaded from Ribosomal Protein Gene Database (http://ribosome.med.miyazaki-u.ac.jp) [41]. For each ribosome protein we used only one RefSeq record. When the mRNA of a ribosome protein was annotated with more than one variant, we considered only those presenting the same extracted sequence in all variants. Eight ribosomal genes with different sequence variants were discarded.

The list of housekeeping genes was taken from recent literature [25]. The list comprises a selection of 67 genes that were common in at least 13 of 15 different lists downloaded from the public domain. Five housekeeping genes were discarded because of different stop codons detected in their CCDS variants.

### Data processing and analysis

Data were principally processed using software programs written in our laboratory in C#, which were tested by independent computational tools and manual calculations. Our software includes programs for sequence and data shuffling using the Fisher–Yates algorithm [42]. Statistical analysis was performed using the software STATISTICA (version 8.0, Statsoft, Inc.) and statistical tools in the software environment R (https://www.r-project.org).

GC-content of CDSs was generally computed considering all codons. In correlation analyses between codon usage and GC content, the results changed very slightly when the codons of the investigated synonymous family were excluded from the calculation of GC-content.

The statistical analysis of stop codon frequencies in GO categories was performed using two statistical models in the software environment R: the pbinom package for the cumulative probability function of binomial distribution and the poibin package [43] for Poisson binomial cumulative probability. In the case of the binomial model, the p-value for the frequency of each stop codon inside the GO categories was determined using the probability estimated from the global genomic codon fraction (p_(UAA)_ = 0.281, p_(UAG)_ = 0.218, and p_(UGA)_ = 0.501). To verify the hypothesis that stop codons are not clustered in GO categories and that the occurrence of a stop codon in a gene is a Bernoulli random variable only related to GC content, we used the Poisson binomial model. The probability p of codon occurrence in each gene was set equal to the codon fraction expected at GC-content of the CDS. The expected codon fraction at each GC-content was computed by a cubic-spline interpolation of real data plotted versus bin of GC-content. In order to simulate a dataset in which codons were randomly distributed among GO categories, the protein identities assigned to each native gene were randomly reassigned. The procedure was repeated 100 times producing 100 sets of the native sequences randomly renamed. The new simulated datasets were used to compute the p-value distribution of stop codon frequencies in the GO categories of a random model. Since the results from binomial and Poisson binomial models were comparable, showing a weak effect of GC-content on p-values, the analysis of the shuffled datasets was approximated using the simpler binomial distribution. The comparisons of p-value distributions in native and shuffled datasets were consistent with a non-random distribution of stop codons in the GO categories.

The MFE of RNA sequences was computed using the RNAfold program included in the ViennaRNA software package version 2.1.9 [44]. To average the contribution of nucleotide order in MFE analyses, we performed 100 shuffles of RNA sequences of all transcript variants and for each gene we used the average MFE of all shuffled variants.

### Ethics approval and consent to participate

Not applicable.

### Consent for publication

Not applicable.

### Availability of data and material

Our software employed to shuffle sequences using the Fisher–Yates algorithm is available through the sourceforge site (https://sourceforge.net/projects/shufflefastaseq).

## Additional files

Additional file 1: Table S1. The twenty GO categories with the strongest deviations from the stop codon frequencies expected on the basis of Poisson binomial cumulative distribution. The probability p of codon occurrence in each gene was set equal to the codon fraction expected at GC-content of the CDS. For binomial distribution the probability p(UAA) was estimated from the relative codon fraction in genome (p(UAA) = 0.2814). (PDF 98 kb)

## Abbreviations

3’ UTR: 3’ untranslated region
GC3: GC content of the third base position of codons
CDS: coding sequence
GO: gene ontology
Barcode: Gene Expression Barcode
Pr: proportion index of Gene Expression Barcode
MFE: minimum folding energy
