# Supplementary material for: Selective forces and mutational biases drive stop codon usage in the human genome: a comparison with sense codon usage
Source: BMC Genomics. 2016 May 17;17:366. doi: 10.1186/s12864-016-2692-4 (PMC4869280; doi:10.1186/s12864-016-2692-4)
Supplement: Additional file 1: Table S1. — The twenty GO categories with the strongest deviations from the stop codon frequencies expected on the basis of Poisson binomial cumulative distribution. The probability p of codon occurrence in each gene was set equal to the codon fraction expected at GC-content of the CDS. For binomial distribution the probability p(UAA) was estimated from the relative codon fraction in genome (p(UAA) = 0.2814). (PDF 98 kb) [file 12864_2016_2692_MOESM1_ESM.pdf]

**Supplementary Table S1.**
**The twenty GO categories with the strongest deviations from the stop codon frequencies expected on the basis of Poisson binomial cumulative distribution. The probability p of codon occurrence in each gene was set equal to the codon fraction expected at GC-content of the CDS. For binomial distribution the probability p(UAA) was estimated from the relative codon fraction in genome (p(UAA) = 0.2814).**

| ID | Name | Ontology | Definition | UAA frequency | UAG frequency | UGA frequency | Number of stop codons | Poissom binomial model P(X≥n. UAA) | Binomial model P(X≥n. UAA) | UAA fraction | UAA fraction without ribosomal proteins |
|---|---|---|---|---|---|---|---|---|---|---|---|
| **GO:0005198** | structural molecule activity | Molecular Function | The action of a molecule that contributes to the structural integrity of a complex or assembly within or outside a cell. | 225 | 114 | 216 | 555 | 5.11E-15 | 2.35E-10 | 0.41 | 0.37 |
| **GO:0045047** | protein targeting to ER | Biological Process | The process of directing proteins towards the endoplasmic reticulum (ER) using signals contained within the protein. One common mechanism uses a 16- to 30-residue signal sequence, typically located at the N-terminus of the protein and containing positively charged amino acids followed by a continuous stretch of hydrophobic residues, which directs the ribosome to the ER membrane and initiates transport of the growing polypeptide across the ER membrane. | 62 | 16 | 25 | 103 | 2.20E-10 | 1.18E-11 | 0.60 | 0.46 |
| **GO:0022626** | cytosolic ribosome | Cellular Component | A ribosome located in the cytosol. | 56 | 14 | 22 | 92 | 2.21E-10 | 6.16E-11 | 0.61 | - |
| **GO:0072599** | establishment of protein localization to endoplasmic reticulum | Biological Process | The directed movement of a protein to a specific location in the endoplasmic reticulum. | 63 | 16 | 28 | 107 | 9.93E-10 | 3.12E-11 | 0.59 | 0.43 |
| **GO:0022411** | cellular component disassembly | Biological Process | A cellular process that results in the breakdown of a cellular component. | 186 | 77 | 186 | 449 | 1.48E-09 | 1.08E-09 | 0.41 | 0.37 |
| **GO:0006614** | SRP-dependent cotranslational protein targeting to membrane | Biological Process | The targeting of proteins to a membrane that occurs during translation and is dependent upon two key components, the signal-recognition particle (SRP) and the SRP receptor. SRP is a cytosolic particle that transiently binds to the endoplasmic reticulum (ER) signal sequence in a nascent protein, to the large ribosomal unit, and to the SRP receptor in the ER membrane. | 59 | 16 | 25 | 100 | 1.52E-09 | 1.16E-10 | 0.59 | 0.39 |
| **GO:0006613** | cotranslational protein targeting to membrane | Biological Process | The targeting of proteins to a membrane that occurs during translation. The transport of most secretory proteins, particularly those with more than 100 amino acids, into the endoplasmic reticulum lumen occurs in this manner, as does the import of some proteins into mitochondria. | 60 | 16 | 26 | 102 | 1.77E-09 | 9.58E-11 | 0.59 | 0.40 |
| **GO:0019083** | viral transcription | Biological Process | The process by which a viral genome, or part of a viral genome, is transcribed within the host cell. | 61 | 16 | 27 | 104 | 2.02E-09 | 7.93E-11 | 0.59 | 0.41 |
| **GO:0070972** | protein localization to endoplasmic reticulum | Biological Process | A process in which a protein is transported to, or maintained in, a location within the endoplasmic reticulum. | 69 | 20 | 36 | 125 | 3.98E-09 | 2.07E-10 | 0.55 | 0.40 |
| **GO:0043241** | protein complex disassembly | Biological Process | The disaggregation of a protein complex into its constituent components. Protein complexes may have other associated non-protein prosthetic groups, such as nucleic acids, metal ions or carbohydrate groups. | 98 | 34 | 75 | 207 | 8.16E-09 | 3.64E-09 | 0.47 | 0.37 |
| **GO:0044391** | ribosomal subunit | Cellular Component | Either of the two subunits of a ribosome: the ribosomal large subunit or the ribosomal small subunit. | 70 | 24 | 40 | 134 | 1.01E-08 | 3.72E-09 | 0.52 | - |
| **GO:0006413** | translational initiation | Biological Process | The process preceding formation of the peptide bond between the first two amino acids of a protein. This includes the formation of a complex of the ribosome, mRNA, and an initiation complex that contains the first aminoacyl-tRNA. | 102 | 29 | 79 | 210 | 1.05E-08 | 3.00E-10 | 0.49 | 0.41 |
| **GO:0043624** | cellular protein complex disassembly | Biological Process | The disaggregation of a protein complex into its constituent components, occurring at the level of an individual cell. Protein complexes may have other associated non-protein prosthetic groups, such as nucleic acids, metal ions or carbohydrate groups. | 91 | 31 | 65 | 187 | 1.12E-08 | 2.31E-09 | 0.49 | 0.37 |
| **GO:0032984** | macromolecular complex disassembly | Biological Process | The disaggregation of a macromolecular complex into its constituent components. | 101 | 34 | 79 | 214 | 1.15E-08 | 2.62E-09 | 0.47 | 0.38 |
| **GO:0019080** | viral gene expression | Biological Process | A process by which a viral gene is converted into a mature gene product or products (proteins or RNA). This includes viral transcription, processing to produce a mature RNA product, and viral translation. | 63 | 20 | 31 | 114 | 1.17E-08 | 1.16E-09 | 0.55 | 0.35 |
| **GO:0003735** | structural constituent of ribosome | Molecular Function | The action of a molecule that contributes to the structural integrity of the ribosome. | 72 | 21 | 46 | 139 | 1.27E-08 | 3.63E-09 | 0.52 | 0.35 |
| **GO:0000184** | nuclear-transcribed mRNA catabolic process, nonsense-mediated decay | Biological Process | The nonsense-mediated decay pathway for nuclear-transcribed mRNAs degrades mRNAs in which an amino-acid codon has changed to a nonsense codon; this prevents the translation of such mRNAs into truncated, and potentially harmful, proteins. | 61 | 14 | 35 | 110 | 1.57E-08 | 1.76E-09 | 0.55 | 0.33 |
| **GO:0022625** | cytosolic large ribosomal subunit | Cellular Component | The large subunit of a ribosome located in the cytosol. | 34 | 7 | 9 | 50 | 1.71E-08 | 5.72E-09 | 0.68 | - |
| **GO:0006415** | translational termination | Biological Process | The process resulting in the release of a polypeptide chain from the ribosome, usually in response to a termination codon (UAA, UAG, or UGA in the universal genetic code). | 80 | 27 | 54 | 161 | 2.38E-08 | 6.24E-09 | 0.50 | 0.34 |
| **GO:0044033** | multi-organism metabolic process | Biological Process | A metabolic process - chemical reactions and pathways, including anabolism and catabolism, by which living organisms transform chemical substances - which involves more than one organism. | 64 | 21 | 35 | 120 | 4.80E-08 | 5.83E-09 | 0.53 | 0.33 |
